# "Floating arm" injury in a child with fractures of the proximal and distal parts of the humerus: a case report

**DOI:** 10.4076/1752-1947-3-9287

**Published:** 2009-09-17

**Authors:** Melih Güven, Budak Akman, Tanzer Kormaz, Oğuz Poyanlı, Faik Altıntaş

**Affiliations:** 1 Department of Orthopaedics and Traumatology, The Hospital of University of Abant Izzet Baysal, Bolu, Turkey; 2 Department of Orthopaedics and Traumatology, Göztepe Training and Research Hospital, Istanbul, Turkey; 3 Department of Emergency Medicine, The Hospital of University of Abant Izzet Baysal, Bolu, Turkey; 4 Department of Orthopaedics and Traumatology, Yeditepe University, School of Medicine, Istanbul, Turkey

## Abstract

**Introduction:**

Simultaneous supracondylar humerus fracture and ipsilateral fracture of the proximal humerus in children is rare.

**Case presentation:**

A 10-year-old Turkish boy with an extension type supracondylar humerus fracture and ipsilateral fracture at the proximal metaphyseal-diaphyseal junction of the humerus was treated by closed reduction and percutaneous Kirschner wire fixation. Closed reduction was performed using a Kirschner wire as a "joystick" to manipulate the humeral shaft after some swelling occurred around the elbow and shoulder.

**Conclusion:**

The combination of fractures at the proximal and distal parts of the humerus can be termed as "floating arm" injury. Initial treatment of this unusual injury should be focused on the supracondylar humerus fracture. However, closed reduction can be difficult to perform with the swelling around the elbow and shoulder. A temporary Kirschner wire can be used as a "joystick" to fix and reduce the fracture.

## Introduction

Supracondylar humerus fractures are usually isolated injuries in children, but sometimes they can be associated with ipsilateral fractures of the forearm. The combination of such injuries is known as "floating elbow" [1]-[4]. However the combination of supracondylar humerus fracture with an ipsilateral fracture of the proximal humerus is extremely rare. To the best of our knowledge, only two cases have been reported in the literature previously [5,6]. It was pointed out in these reports that swelling around the elbow and shoulder regions could make closed reduction difficult. We describe the case of a 10-year-old boy who had an extension type supracondylar humerus fracture and ipsilateral fracture at the proximal metaphyseal-diaphyseal junction of the humerus. Both fractures were treated successfully by closed reduction and percutaneous Kirschner wire fixation.

## Case presentation

**Figure 1:**
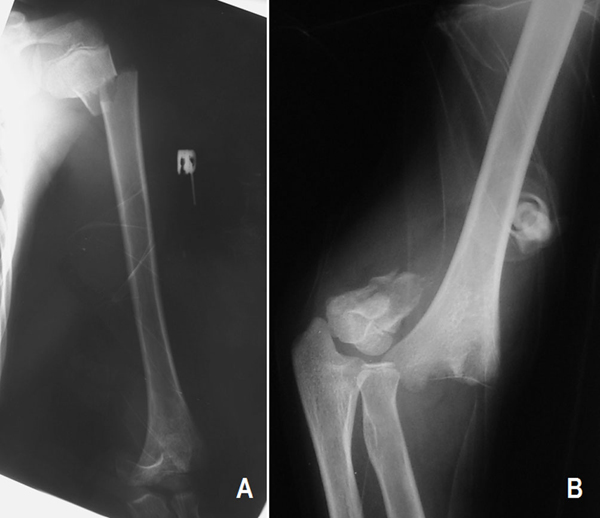
**Preoperative **(A)** anteroposterior roentgenogram of the left upper extremity and **(B)** lateral roentgenogram of the left elbow**.

A 10-year-old Turkish boy who had fallen from a height of approximately 2 meters onto his outstretched left hand was referred to our hospital one hour after the injury. He complained of pain in the left elbow and shoulder. The proximal part of the left arm and elbow had swelling and crepitus. His neurovascular examination was normal. Plain radiographs showed a displaced fracture at the proximal metaphyseal-diaphyseal junction of the left humerus and ipsilateral displaced extension type supracondylar humerus fracture (Figure 1). His left upper extremity was splinted and the patient was taken to the operating room on the same day.

**Figure 2:**
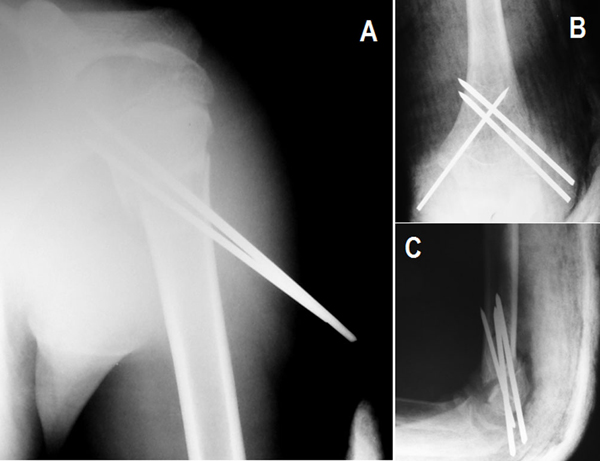
**Early postoperative roentgenograms of **(A)** left shoulder and **(B, C)** left elbow**.

Under general anesthesia, closed reduction of the supracondylar humerus fracture was initially attempted under fluoroscopic control. However, it could not be achieved with manipulation because it was difficult to perform closed reduction on the severely swollen upper limb. A 3 mm temporary Kirschner wire was inserted to the humeral shaft from the lateral to medial direction. With the assistance of this "joystick" Kirschner wire, the humeral shaft was manipulated easily and closed reduction and percutaneous fixation with three Kirschner wires were performed. Once the supracondylar humerus fracture was stabilized, the management of the proximal humerus fracture was relatively straightforward. Closed reduction with gentle manipulation and percutaneous fixation with two Kirschner wires were applied to the proximal humerus fracture (Figure 2). After the operation, we used a plaster of Paris splint to immobilize the left upper extremity.

**Figure 3:**
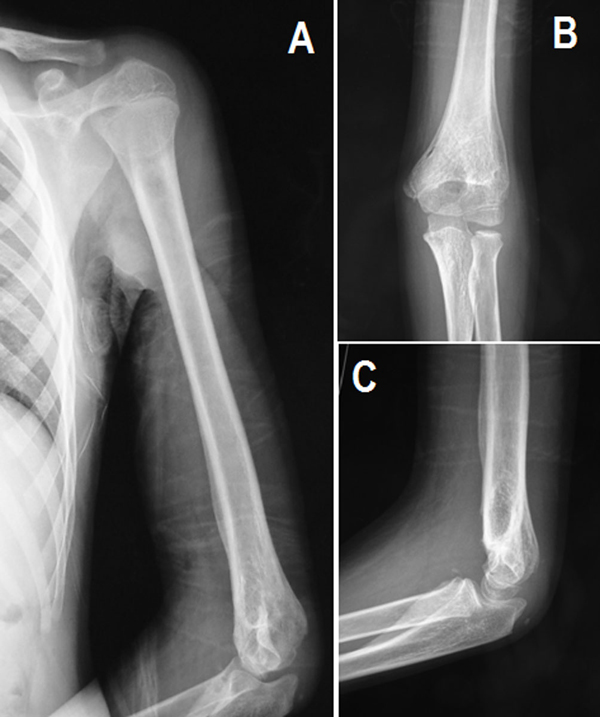
**Postoperative roentgenograms of **(A)** left upper extremity and **(B, C)** left elbow at six months**.

We removed the Kirschner wires from both the elbow and the shoulder and the splint as an outpatient procedure after 4 weeks of radiographic healing and a range of motion exercises were begun. During the final follow-up 6 months after the surgery, plain radiographs showed adequate healing without any angular deformity on the elbow and shoulder (Figure 3). The patient gained full function of shoulder motion, with elbow extension and/or flexion of 0° to 130°, pronation and/or supination of 80° each and equal to the uninjured side.

## Discussion

Stanitski and Micheli [1] first used the descriptive term "floating elbow" to describe the combination of ipsilateral fracture of the elbow and forearm. Gausepohl *et al.*[7] reported a case with fracture dislocation of the elbow combined with unstable distal forearm fracture of the ipsilateral upper extremity and termed this injury as "floating forearm". Similarly, we prefer the term "floating arm" to describe this rare combination of the fractures at the elbow and shoulder regions.

We have found only two cases in the English language literature in which the combination of ipsilateral proximal humerus fracture, flexion type supracondylar humerus fracture and olecranon fracture were present [5,6]. The authors of both reports recommended that the supracondylar humerus fracture should be reduced first and percutaneously fixed before the reduction of proximal humerus fracture. However, they could not achieve closed reduction for the supracondylar humerus fracture due to the combination of fractures in the same extremity and resulting instability. Therefore, they performed open reduction.

Extension types of supracondylar humerus fractures are mostly accepted as pure hyperextension injuries that are caused by a fall onto the outstretched hand with hyperextension of the elbow [8]. Proximal humerus fractures in children can occur as a result of a direct blow to the shoulder area or indirectly as a fall onto an outstretched hand. This causes a forced position of the upper extremity resulting in a fracture of the proximal humerus [9]. The "floating arm" injury presents a more serious injury than an isolated supracondylar fracture or an isolated fracture of the proximal humerus and reflects a more violent episode of trauma. Due to swelling around the elbow and shoulder, closed reduction, especially for the supracondylar humerus fracture, is not always possible. Parmaksizoglu *et al.*[10] described an alternative closed reduction method to avoid open reduction for supracondylar humerus fractures in children. They concluded that a temporary Kirschner wire driven as a "joystick" to the humeral shaft before percutaneous fixation made reduction and fixation of the supracondylar humerus fracture easier by controlling the proximal fragment. This technical trick also facilitates closed reduction of the supracondylar humerus fracture in a "floating arm" injury like in our case. A temporary Kirschner wire allows the surgeon to stabilize the humeral shaft and control the motion in the coronal, sagittal and horizontal planes for both supracondylar and proximal humeral fractures.

## Conclusion

The supracondylar humerus fracture should be reduced initially in a "floating arm" injury. However, closed reduction can be difficult to perform due to severe swelling around the elbow and shoulder regions associated with this injury. In such cases, a temporary Kirschner wire can be used as a "joystick".

## Competing interests

The authors declare that they have no competing interests.

## Consent

Written informed consent was obtained from the patient's parents for publication of this case report and any accompanying images. A copy of the written consent is available for review by the Editor-in-Chief of this journal.

## Authors' contributions

MG and BA contributed to the conception and design, and carried out the literature research, manuscript preparation and manuscript review. TK and OP were involved in the literature review and helped draft part of the manuscript. FA contributed to the conception and design. MG, TK and OP revised the manuscript.
